# Potential Serum Biomarkers in Prenatal Diagnosis of Placenta Accreta Spectrum

**DOI:** 10.3389/fmed.2022.860186

**Published:** 2022-05-30

**Authors:** Tianyue Zhang, Shaowei Wang

**Affiliations:** ^1^Department of Gynaecology and Obstetrics, National Center of Gerontology, Beijing Hospital, Beijing, China; ^2^Institute of Geriatric Medicine, Chinese Academy of Medical Sciences, Beijing, China; ^3^Graduate School of Peking Union Medical College, Beijing, China

**Keywords:** biomarker, prenatal diagnosis, maternal blood, pregnancy, placenta accreta spectrum

## Abstract

Placenta accreta spectrum (PAS) refers to the abnormal invasion of trophoblastic tissues. Because of its increasing morbidity and possibility of catastrophic outcomes, PAS requires an antenatal diagnosis and making full preparations in advance to realize safe delivery. Current clinical screening modalities for PAS are not always conclusive. Recently, it has been reported that bio-markers detected in maternal serum have the potential for predicting PAS during pregnancy. Some of these biomarkers, such as β-hcg, AFP, PAPP-A, and cffDNA, can be clinically detected. It is convenient for us to test and compare with standard threshold. However, how can we distinguishing PAS from other pregnancy complications through these biomarkers remains complicated. Some biomarkers are specific, such as microRNA and placenta-specific mRNA. They are stability and reliability. These biomarkers are currently research hotspots. This study aims to summarize the characteristics of the newly reported biomarkers and to point out their potential application and current limitations to provide a basis for future research. Finally, the combination of imageological examination and biomarkers will be an attractive future theme to study in diagnosing this challenging condition.

## Introduction

Placenta accreta spectrum (PAS) refers to the penetration of trophoblastic tissues through the decidua basalis into the uterine myometrium, uterine serosa or beyond, extending to the parametrium or the adjacent pelvic organs. PAS is classified depending on the degree of invasion, as placenta adherenta or creta (PC), placenta increta (PI), or placenta percreta (PP) (1). The prevalence of PAS in studies published between 1982 and 2018 ranged from 1 in 100 to 1 in 10,000. The incidence of PAS has been increasing over the past several decades, and can be attributed to the history of cesarean section (CS), previous placenta accreta, placenta previa, maternal age, IVF pregnancies, and Maternal obesity (2, 3).

Women with placenta increta and percreta have a considerably high risk of surgical morbidity and mortality. The adverse pregnancy outcomes (APOs) caused by PAS including the following:(i) Hemorrhage is one of the major complications, and the surgical blood loss could be up to several liters, possibly leading to disseminated intravascular coagulation (DIC), shock, and even death. (ii) Hysterectomy and urinary tract injury are other common outcomes, which may have a profound influence on women's long-term life quality. All these terrible outcomes harm both the women's mental and physical health (4).

An antenatal diagnosis of PAS and identifying the type of PAS can provide time for preoperative management. Preoperative coordination with protocol-based interdisciplinary care could optimize intraoperative and postoperative outcomes. Being fully prepared with enough blood, hemostatic and oxytocic drugs, prophylactic positioning of balloon vascular catheters, placement of ureteric stents preoperatively, and autologous cell salvage equipment can also reduce APOs and minimize manual interventions (5, 6). In the UK, a cohort study demonstrated that antenatal diagnosis was associated with reduced levels of hemorrhage (median estimated blood loss 2,750 vs. 6,100 ml, *p* = 0.008) and a reduced need for blood transfusions (59 vs. 94%, *p* = 0.014) (7).

In recent years, it has been reported that a variety of biomarkers are associated with PAS. Because ultrasound and MRI do not provide a sufficient diagnosis for PAS, biomarkers of PAS might be clinically useful. This review aims to discuss some of the recent advances in these biomarkers.

## Biomarkers

### Alpha-Fetoprotein (AFP)

Maternal serum alpha-fetoprotein (MS-AFP) is a glycoprotein comprising an α-globulin of 591 amino acids and a partial carbohydrate. MS-AFP is synthesized by the yolk sac during early pregnancy, and later by the fetal gastrointestinal tract and liver, which is the main source throughout the rest of the pregnancy. AFP is transported to maternal serum through the placenta or by diffusion across fetal membranes. As a result, AFP is the principal serum binding protein in the fetus. As a second-trimester biochemical marker, MS-AFP has been widely applied for prenatal screening in practice. High or low MS-AFP suggests a high risk of fetal open neural tube defects (ONTDs) or chromosomal aneuploidy (8).

In recent years, new researchers found that elevated AFP levels associated with PAS. Because of early placental vascular damage, MS-AFP may be more absorbed into maternal blood flow. A retrospective case-control study in Israel confirmed this viewpoint. Berezowsky et al. observed that each individual PAS group had a higher median MS-AFP of median (MoM) than the controls (*p* = 0.033) in the second trimester. They also calculated that the receiver operating characteristics (ROC) area under the curve (AUC) of the AFP area was 0.573 (95% CI 0.515–0.630, *p* <0.0274) and a cut-off value above 0.99 MoM demonstrated a sensitivity and specificity of 71 and 46%, respectively, for the prediction of PAS (9). Furthermore, increased second trimester MS-AFP levels can also independently predict PAS requiring hysterectomy among women with placenta previa totalis in a previous study. In a retrospective review of 316 pregnant women whom identified as cases of placenta previa totalis, Oztas et al. found that MS-AFP levels were significantly higher in second-trimester screenings of patients with PAS requiring hysterectomy (*p* < 0.001). The predictive AUC of ROC was 0.742 (95% CI: 0.505–0.979). The best MS-AFP cut-off value was 1.25 Mom, with 85.94% sensitivity and 71.43% specificity (*p* = 0.036). A multivariate logistic regression analysis was carried out to verify that MS-AFP was an independent predictor of PAS requiring hysterectomy [odds ratio (OR) = 25.329, 95% CI:1.487–43.143, *p* = 0.025] (10).

AFP is wildly applied in clinical treatments, including monitoring specific types of cancer and screening for fetal malformation. This implies that AFP testing methods are mature and its price is affordable, which makes screening for PAS convenience. However, even though MS-AFP has been shown to be a predictable factor of PAS in the second trimester and can also independently predict PAS requiring hysterectomy, the following restrictions remain: (i) Until now, few articles have demonstrated the relationship between MS-AFP and PAS, and this relationship's reliability and accuracy are insufficient. (ii) Whether MS-AFP can predict PAS in the first trimester or whether MS-AFP can predict other PAS-associated complications remains unknown. (iii) In addition, the cut-off value of MS-AFP to distinguish PAS varied across studies; thus, further prospective studies with larger populations are needed to find an exact cut-off value. (iv) Because MS-AFP is influenced by several factors, including fetal abnormalities and other types of placental abnormalities, these factors must be considered in future studies to reduce bias. Therefore, significant work remains to apply MS-AFP in practice to predict PAS.

### Human Chorionic Gonadotropin (hCG)

A glycoprotein composed of 244 amino acids and with a molecular mass of 36.7 kDa. hCG is produced by the syncytiotrophoblast and maintains pregnancy by stimulating progesterone synthesis by the corpus luteum. Furthermore, HCG is a heterodimeric molecule composed of an alpha subunit that is identical to several hormones and a beta (β) subunit that is unique. In normal pregnancies, maternal circulating concentrations of ß-hCG show an increase from 9 to 11 weeks of gestation, followed by a decline thereafter. β-hCG is also synthesized by the fetal kidney and fetal liver. Free β-hCG promotes angiogenesis, and cytotrophoblast differentiation, and blocks the phagocytosis of invading trophoblastic cells, immunosuppression and blocks the phagocytosis of invading trophoblast cells (11).

Research has shown that β-hCG maternal serum levels are differential in cases of PAS during gestation. A team in Taiwan first reported this phenomenon in 1999, noting that abnormally elevated second-trimester free β-hCG levels were a risk factor for placenta accrete, and placenta previa (11). In 2015, in a prospective observational cohort study, Thompson et al. obtained maternal serum of 516 pregnancies, including 344 normal controls, 17 with abnormally invasive placentation (AIP) and 155 placenta previa cases in the first trimester. They demonstrated a median free β-hCG MoM of 1.04 in the control group, and 1.08 (*p* = 0.859) in the placenta previa group compared to 0.81 in the AIP group (*p* = 0.06), which suggested the relationship between PAS and β-hCG (12). Another study in Turkey arrived at the same conclusion. When Buke et al. found that patients with placenta accreta had higher statistically significant serum fβ-hCG MoM (1.42 vs. 0.93, respectively, *p* = 0.042) values than patients without accreta in the first trimester (13). In another study, which evaluated the association between second trimester biochemical markers and pathological placentation for hCG, the AUC was 0.662 (95% CI 0.605–0.715, *p* < 0.0001), and the cut-off value of 1.25 MoM demonstrated a sensitivity and specificity of 53 and 68%, respectively (9). All of these studies confirmed the association between β-hCG and PAS. Currently, serum β-hCG levels are wildly used in clinical practice, including identifying the pregnancy, monitoring embryo changes, suggesting certain types of cancer etc. Thus, MS-AFP and β-hCG are all excellent options for prenatal diagnosis PAS in the future. There remain some difficulties that must be solved. For instance, β-hCG levels fluctuate wildly during the first trimester, reaching up to 100,000 U/ml. This variation trend in hCG must be further understood to choose the appropriate cut-off value to predict PAS. Furthermore, serum hCG levels are also associated with some other conditions, including miscarriage, ectopic pregnancy, and fetal abnormalities. It is difficult to distinguish the maternal serum hCG levels of PAS from those other conditions. Therefore, these difficulties must be overcome to make detecting biomarkers feasible in the future.

### Pregnancy-Associated Plasma Protein A (PAPP-A)

PAPP-A, a zinc metalloproteinase produced by placental syncytiotrophoblasts, is secreted into the maternal circulation in increasing concentrations until delivery. Although its function is not well-understood, PAPP-A is commonly recognized as being responsible for proteolysis of insulin-like growth factor (IGF) from IGF-binding protein-4(IGFBP-4), implying a role in growth. In previous studies, scientists have demonstrated that low PAPP-A has been associated with poor placental invasion, resulting in placental insufficiency, intrauterine growth restriction, preeclampsia, stillbirth, abruption, and premature birth etc. Based on these findings, PAPP-A may play a significant role in regulating trophoblastic invasion (12, 14).

Owing to trophoblastic abnormal invasion, PAS's association with PAPP-A was first reported in 2014, when PAPP-A was found to be elevated during the first trimester in cases of PAS. The median PAPP-A MoM of 1.68 in accreta was significantly >0.98 in non-accreta (*p* = 0.002) (11). In a subsequent study, several researchers confirmed this conclusion. In 2015, Thompson et al. provided an overall PAPP-A median MoM of 1.40 (*p* = 0.002) in the first trimester by analyzing an AIP dataset from London and New York (12). In the same year, Lyell et al. also confirmed that elevated PAPP-A was associated with PAS among women with previa in the first trimester (14). However, while serum PAPP-A cannot be a diagnostic marker because there is significant overlap with the distribution of unaffected pregnancies, it can be used as a tool to modify the risk of placenta accreta. Furthermore, as a PAS biomarkers, serum PAPP-A can also predict the prognosis of PAS. Penzhoyan et al. found a significant correlation (*p* <0.15) between the PAPP-A and the blood loss volume (PAPP-P M?M was 1.37 ± 1.20 in the not higher than 1,000 mL (LBL) subgroup and 1.91 ± 1.24 in the higher than 1,000 mL (HBL) subgroup MoM indicators, *p* = 0.091). This research revealed that the PAPP-A level in the first trimester may be helpful for the early prognosis of pathological blood loss at delivery for pregnant women with PAS and for forming a high-risk group for PAS (15). Hence, PAPP-A plays an important role in identifying high-risk population for PAS. Based on the levels of measurement technology, it is easy for researchers to test maternal-serum PAPP-A. Because PAPP-A isn't an independent risk factor for PAS, a model must be created to predict the risk of PAS. Previous research studies have presented prediction models. Research bottlenecks include finding proper influential factors and building an optimal model are research bottlenecks. To break these bottlenecks, bioinformatics specialists must participate in the research.

### Angiogenic Factor

When abnormal placentation results in PAS, the process of excessive neovascularization occurs and upregulates angiogenic factors, such as vascular endothelial growth factor (VEGF) and angiopoietin 2 (Ang-2), and downregulates anti angiogenic factors, such as soluble Fms-like tyrosine kinase 1 (sFlt-1) and VEGFR-2 in syncytiotrophoblast. Accordingly, placental growth factor (PLGF), a high-affinity binder to VEGFR-2, is increased in serum levels. Researchers have quantified all these serum factors using enzyme-linked immunosorbent assays (ELISAs). Thus, when PAS happens, maternal serum angiogenesis factors could change. In the third trimester, women with PAS have significantly lower maternal serum VEGF levels because oxidative stress might lead to a down regulation of VEGF, and PLGF and sFlt-1 were significantly higher in PAS. Thus, these indicators can help predict PAS. Furthermore, in a multicenter case-control study design in Germany, Schwikert et al. collected gestational age-matched serum samples from pregnant women with AIP (*n* = 44) and uncomplicated pregnancies (*n* = 55) before delivery (GA median: 35 weeks). They concluded that maternal serum VEGF levels exhibited a significantly negative correlation with the FIGO AIP degree of invasion (Spearman'srho = −0.37; *p* < 0.001). Serum VEGF was able to predict the need for peripartum hysterectomy by plotting RUC curves (AUC = 0.698, 95% CI 0.564–0.832, LR+2.4, LR−0.3, *p* = 0.004). This phenomenon provides a clear advantage for clinical application (16–18). However, due to the study's sample sizes and limited relevant reports, these remain controversial conclusions. In researching angiogentic factors, a 2016 case–control study in Turkey demonstrated that there was no statistical difference in the maternal serum values of sFlt1, PlGF, sFlt1/PlGF ratio, and VEGF in groups with placental abnormalities compared to controls (19). However, these findings were not confirmed in subsequent studies.

In summary, the reviewed research studies indicate that PLGF, VEGF, and sFlt-1 have great application prospects in the future, although research on them is limited. Future research must focus on influential factors, such as diabetes, hypertension, pulmonary and cardiovascular disorders, FGR, inflammatory or connective tissue disorders, liver or renal failure, and placenta separation. How to rule out the effects of these factors and choose an appropriate cut-off value for them must still be resolved. Furthermore, the reviewed studies were all performed in the third trimesters. Whether such results could be acquired from samples taken during other trimesters remains unknown.

### Cell-Free Fetal DNA (cffDNA)

In 1997, Y M Dennis Lo etc. first reported that cffDNA was present in maternal plasma and serum, and it is believed to originate from the apoptosis of cytotrophoblast and syncytiotrophoblast cells (20). It is generally recognized that these cells are destroyed by the maternal immune system during the implantation process. In a previous study, cffDNA occurred during pregnancy and rapidly cleared no more than 2 h after delivery. Gradually, the finding's applicating became widespread in clinical practice, aiming to find chromosomal aneuploidies. The level of cffDNA has also been shown to be associated with some pregnancy pathologies, including preterm birth, fetal growth restriction, and preeclampsia.

In 2002, a study verified that the concentration of fetal DNA within maternal plasma was significantly increased in the third trimester of two cases of invasive placentas (20). Since then, scientists have realized that cffDNA is related to PAS. In 2003, Jimbo et al. applied plasma cffDNA in PI's postpartum monitoring. After a gestation of 37 weeks and 3 days, a pregnant woman delivered and was diagnosed with PI through her clinical symptoms and magnetic resonance imaging findings. A small part of the placenta (7 cm in diameter) could not be removed from her uterus. The doctors monitored serum cffDNA regularly. The patient had intermittent vaginal bleeding until fetal DNA (DYS14 DNA) could no longer be detected in her plasma around 10 weeks post-delivery. The remaining portion of the placenta was spontaneously delivered 10 weeks postpartum. This case indicated that cffDNA in the plasma of pregnant women might serve as a molecular marker for the detection of AIP. In 2020, another case reported that a patient with AIP had a highly elevated level of cffDNA fraction (35.3%) at the end of the first trimester, whereas the median level of cffDNA fraction in normal pregnancies at this gestational age was only around 10.4%. The cffDNA fraction then decreased to 26.1% at 26 + 6 weeks of gestation (the median level at this gestational age was around 18%, according to a previous report) (21). Therefore, this supports the assumption that the cffDNA fraction may be a better biomarker for PAS. However, there are still some contradictory results to these findings. In 2013, a pilot study was carried out with 20 women [seven cases of placenta accreta, six cases of placenta previa and seven cases of normal placentation with prior cesarean deliveries (CD)]. After qualifying maternal serum cffDNA levels in the third trimester, the mean fraction of cffDNA prior to delivery did not differ significantly by group (accreta = 19.1%, previa = 27.2%, prior CD = 28.9%, *p* = 0.26), nor did the median (accreta = 17.0%, previa = 30.1%, prior CD = 22.7%), after controlling for no significant difference in maternal weight, placental weight, number of prior cesarean deliveries, or years from prior cesarean delivery (20). However, it may also be possible that the PAS process occurs much earlier in gestation, and it has been suggested that a significant difference between groups could be seen during the first trimester. Thus, from above study, the definite relationship between cffDNA and PAS is unclear. Since cffDNA is stable and can be detected in the early trimester, it can be an appropriate bio-marker. However, serum cffDNA samples are easily influenced by many factors, including their concentrations, the technique for isolation of DNA, gestation of the acquired sample, and length range of cffDNA. Further research must be performed in consideration of these factors. The number of samples in the current study is still too low. A larger clinical cohort trial of cffDNA is expected to be conducted in the future. In conclusion, the testing of maternal serum cffDNA for PAS is in its early stage. CffDNA as a possible marker of subsequent APS could help to improve the accuracy of the prenatal diagnoses of PAS.

### Placenta-Specific mRNA

The placenta is a major contributor to Cell-free mRNA. Circulating cell-free placental mRNAs in maternal plasma can be stably isolated, quantified, and rapidly cleared after delivery. The mechanism of these actions is still unclear. The mRNA may transfer between the placenta and the maternal circulation due to a thin and dysfunctional decidua (26). Therefore, placenta mRNA could be investigated as a potential markers for placental function and placental-related disorders. Researchers mainly pay attention to two major placenta-specific mRNAs: β-human chorionic gonadotropin (β-hCG) and human placental lactogen (hPL) placental mRNAs.

#### Human Placental Lactogen (hPL) mRNA

During placental formation, trophoblast cells play an important role in placenta invasion into the uterus. Cell-free hPL mRNA is mainly produced in the mature syncytiotrophoblasts. In 2005, Masuzaki et al. demonstrated that a patient with placenta percreta had a residual placental mass at the internal os. They found that hPL mRNA could be used to monitor the efficacy of methotrexate therapy for PP, which suggested placental mRNA may be useful as a predictive marker for PAS (27). In 2014, by comparing the plasma of pregnant women at 28–32 weeks of gestation, Kawashima concluded that expression of hPL mRNA is increased in the plasma of women with invasive placenta (*p* < 0.05) (28). Subsequently, in a case-control study, Jing et al. collected peripheral blood from each woman at 28–30 weeks of gestation and measured maternal plasma hPL mRNA concentrations by real-time reverse-transcription polymerase chain reaction (PCR) in 2019. They came to the same conclusion that the multiple of the median (median, range) for hPL mRNA was significantly higher for the placenta accreta group (2.78, 1.09–4.56) than the control (1.00, 0.29–2.98) or placenta previa (1.12, 0.33–3.25) groups (Steel–Dwass test, *p* < 0.001 and *p* = 0.005, respectively). Furthermore, in 2008, a case-control study demonstrated that the cell-free hPL mRNA concentration in maternal plasma has the potential to predict a subgroup of placenta accreta resulting in hysterectomy; however, the results were not convincing due to the limited simple size (26).

#### β-Human Chorionic Gonadotropin (β-hCG) mRNA

Cell-free β-hCG mRNA is primarily produced by proliferating cytotrophoblasts. In 2005, it was reported that β-hCG mRNA could be used to monitor the efficacy of methotrexate therapy for placenta percreta. Therefore, it may be possible for β-hCG mRNA to predict PAS. Zhou et al. compared β-hCG mRNA concentrations of blood plasma from 68 pregnant women between 28–30 weeks. They reported that cell-free-β-hCG mRNA concentrations (MoM, range) were significantly higher in women with placenta accreta (3.65, 2.78–7.19) than in women with placenta previa (0.94, 0.00–2.97) or normal placentation (1.00, 0.00–2.69; Steele-Dwass test, *p* < 0.01 and *p* < 0.01, respectively). Furthermore, they found that seven women who underwent hysterectomies in the placenta previa/accreta group had higher concentrations of cell-free β-hCG mRNA (4.41, 3.49–7.19) compared with women whose deliveries did not result in hysterectomy (3.20, 2.78–3.70; Manne-Whitney *U*-test, *p* = 0.012), which demonstrates β-hCG mRNA may be related to a prognosis of PAS (29). Currently, with the emergence of real-time polymerase chain reaction (RT-PCR) technology, doctors can easily to monitor placental-specific mRNA levels. Placental-specific mRNAs are not only potential biomarkers to predict PAS, but they also have the possibility to predict APOs. However, large prospective studies remain to be performed to confirm the role of hPL mRNA and cell-free-β-hCG mRNA in predicting AIP and the best time to harvest maternal blood. The technology's cost should be reduced to increase its applicability. The present study did not explore the association of placental-specific mRNA with the degree of PAS. Further studies with subdivisions are needed.

Because the range of mRNA is wider than that of protein, the use of plasma mRNA markers may increase the number of markers that can be used for prenatal monitoring and its greater specificity and sensitivity make the prenatal diagnosis of PAS more exact.

### MicroRNA

MicroRNA, a non-coding RNA of ~22 nucleotides, interferes with mRNA or DNA expression and occurs at the transcriptional and post-transcriptional levels. Circulating miRNAs can be detected in plasma and serum stably, because of their resistance to endogenous ribonuclease activity and can function as vesicles forming exosomes with lipoprotein membranous complexes or clusters. Micro-RNAs have been shown to modulate cell differentiation, embryonic development, adhesion, migration, apoptosis, and angiogenesis during placental development, and the altered expression of miRNA has been associated with various pregnancy complications. According to the mechanism, researchers can obtain a sample of maternal peripheral blood plasma, isolated the RNA from the sample, perform reverse transcription and qRT-PCR, and then conduct a quantitative analysis. Therefore, some studies have revealed that level of microRNA is in accordance with the PAS (Table 1). It is believed that miRNAs may predict PAS. Recently, scientists have also proven that some microRNAs, such as miR-17-5p, miR-21-5p, miR-25-3p, miR-92a-3p, and miR-320a-3, are associated with severity of PAS (23). Researchers also found that miRNA can predict PAS prognosis. In one study, Tian Yang et al. indicated that the expression of hsa-miR-490-3p and hsa-miR-133a-3p was positively correlated with operation-related blood volume loss. Furthermore, another study found that the amount of blood loss was negatively correlated with the expression of miR-139-3 p, and miR-196a-5p, with correlation coefficients of 0.215 and 0.172, respectively (*p* < 0.01 and *p* < 0.05, respectively) (24). For its specify and variety, MicroRNAs have bright prospects for prenatal diagnosis of PAS. At present, more than 20,000 miRNAs can be detected in the placenta; however, only a few miRNAs have been confirmed to be associated with PAS. More research must be conducted to find additional related miRNAs. Recently, miRNA research has become a hotspot. Nearly all of above miRNAs have been mentioned only once in existing research, which indicates very low reliability. More research is needed to find suitable miRNAs in PAS and to verify the finding in larger samples. Another difficulty is that detecting miRNA is a tedious process, which means it comes at a high price. Thus, simplifying process and decreasing price to make miRNA detection affordable will be other important tasks in the future.

**Table 1:** miRNAs in PAS.

**miRNAs**	**Trend**	**Sample**	**Gestational weeks**	**Target signaling molecular**	**Function**	**Ref**
hsa-miR-10524-5p hsa-miR-133a-3p hsa-miR-937-3p hsa-miR-34b-5p hsa-miR-3529-3p hsa-miR-488-3p hsa-miR-490-3p	↑/↓	Peripheral blood plasma	Before cesarean section	–	Angiogenesis and blood vessel morphogensis	(22)
hsa-miR-17-5p hsa-miR-21-5p hsa-miR-25-3p hsa-miR-92a-3p hsa-miR-320a-3p	↑	Peripheral blood plasma	30–34w	Clusterin	Regulate the epithelial- mesenchymal transition, decrease the expression level of clusterin	(23)
miR-196a-5p, miR-518a-3p, miR-139-3p miR-671-3p	↓	Venous blood	third trimester	NF-κB, ANXA1, MMP11, E-cadherin	Angiogenesis, embryonic development, cell migration and adhesion, and tumor-related pathways	(24)
Micro-7	↓	Peripheral blood plasma	–	TGF-β	Promotes trophoblast invasion	(25)

### Others

Researchers have also found other biomarkers related to PAS. Shainker et al. used an aptamer-based proteomics platform to examine the maternal plasma samples from 16 patients with PAS for alterations in 1,305 unique proteins, finding 50 proteins with expression levels that were significantly different (*p* < 0.01). After analyses, the researchers found that the proteins were mostly inflammatory cytokines, factors that regulate vascular remodeling, and extracellular matrix proteins that regulate invasion. All of them may be potential biomarkers for diagnosing PAS and require further study (30). In reality, some proteins with specific functions are reportedly associated with PAS. A prospective case-control study reported that the maternal serum thyroid-stimulating hormone (TSH) and thyroid antibodies against thyroglobulin (TgAb) levels were significantly lower in a PAS group than placenta previa and control groups early in the third trimester. Decreased serum TSH and TgAb levels were also found to be independently associated with an increased rate of cesarean hysterectomy and massive blood transfusions in PAS cases (31). TRAIL-R2 is widely known as a death receptor (DR) and induces apoptotic signaling. Serum TRAIL-R2 levels have been shown to be significantly lower in a PA group (median 19.85 pg/mL) when compared with both non-adherent placenta previa (median 25.99 pg/mL) and control groups (median 25.87 pg/mL; *p* < 0.05). Thus, not only is TRALL-R2 associated with PAS, but it also plays a possible role in apoptosis in PAS (32). In addition to protein, some specific genes may also be related to PAS. However, only one case report found a possible association between placenta accreta and manifestations of the A3243G mutation in mtDNA (33). Other genomic research studies have been performed at the placental level. Hundreds of genes have significantly different detection levels in placental tissues. Whether these genes are the same in peripheral blood is not known. Today, NanoVelcro chips, which are nanostructure-embedded microchips, can efficiently enrich both single and clustered circulating trophoblasts (cTBs) from maternal blood. In a multicenter observational cohort study of 168 pregnant women, the combined cTB assays achieved an area under ROC curves of 0.942 (throughout gestation) and 0.924 (early gestation) for distinguishing PAS from non-PAS. For its simple operation procedure and high accuracy, the combination of cTBs and cTB clusters captured on the NanoVelcro chips for detecting PAS early in gestation will enable a promising quantitative assay to serve as a non-invasive test (34).

### Potential Biomarkers

According to the current study, some biomarkers have been detected to be significantly different in placenta tissues, which may identify them as potential maternal biomarkers to predict PAS. For example, in 2019, to explore lncRNA differential expression, Huishan Zhang et al. screened five pairs of placenta tissues of patients with PAS. Using RNA microarray analysis, a total of 329 lncRNAs (101 upregulated; 228 downregulated) and 179 mRNAs (95 upregulated; 84 downregulated) were identified as having differential expressions in PAS. This makes it easier to select further biomarkers (35). Furthermore, by sequencing the placenta tissues of five women with PAS and five healthy pregnant women, Qiao et al. found that 23 hub genes were differentially expressed at both the mRNA and protein levels and that the expression trends were the same (36). Hub genes and bottleneck genes may have more specificity for detecting PAS. With the progression of omics studies in PAS and increasing sample sizes, the genetic features of PAS will be clearer, and the structural variations of hub genes will be more statistically significant, which will provide potential predictive and therapeutic targets for PAS.

### Current Situation and Prospective Future of PAS

PAS is one of the main causes of increasing maternal morbidity and mortality, with consequences ranging from excessive hemorrhage, the need for hysterectomy, DIC, massive transfusions, ureteral/bladder/bowel injury, and even death. Neonates also have a higher risk of suffering neonatal complications, such as NICU admission and transient tachypnea of newborns (TTN). Considering the risks and APOs among patients with PAS, the significance of the condition and the need for early diagnosis has become more apparent. Antenatal diagnosis could transfer pregnant women to highly experienced maternity centers in time. Furthermore, it allows obstetricians to organize a comprehensive multidisciplinary care team and make full preparations, including advanced surgical planning, abundant blood supplies, preoperative preparations, and appropriate time allotted for delivery. In 2018, the American College of Obstetricians and Gynecologists (ACOG) guidelines for PAS advised that delivery at 34 0/7–35 6/7 weeks of gestation is the preferred gestational age for scheduled cesarean delivery or hysterectomy, absent extenuating circumstances, in a stable patient. Earlier delivery may be required in cases of persistent bleeding, preeclampsia, labor, rupture of membranes, fetal compromise, or developing maternal comorbidities. The guidelines also recommend the use of antenatal corticosteroids for lung maturation in women with antenatally diagnosed PAS (37). All these recommendations attach great importance in prenatal diagnosis PAS.

**Figure 1:**
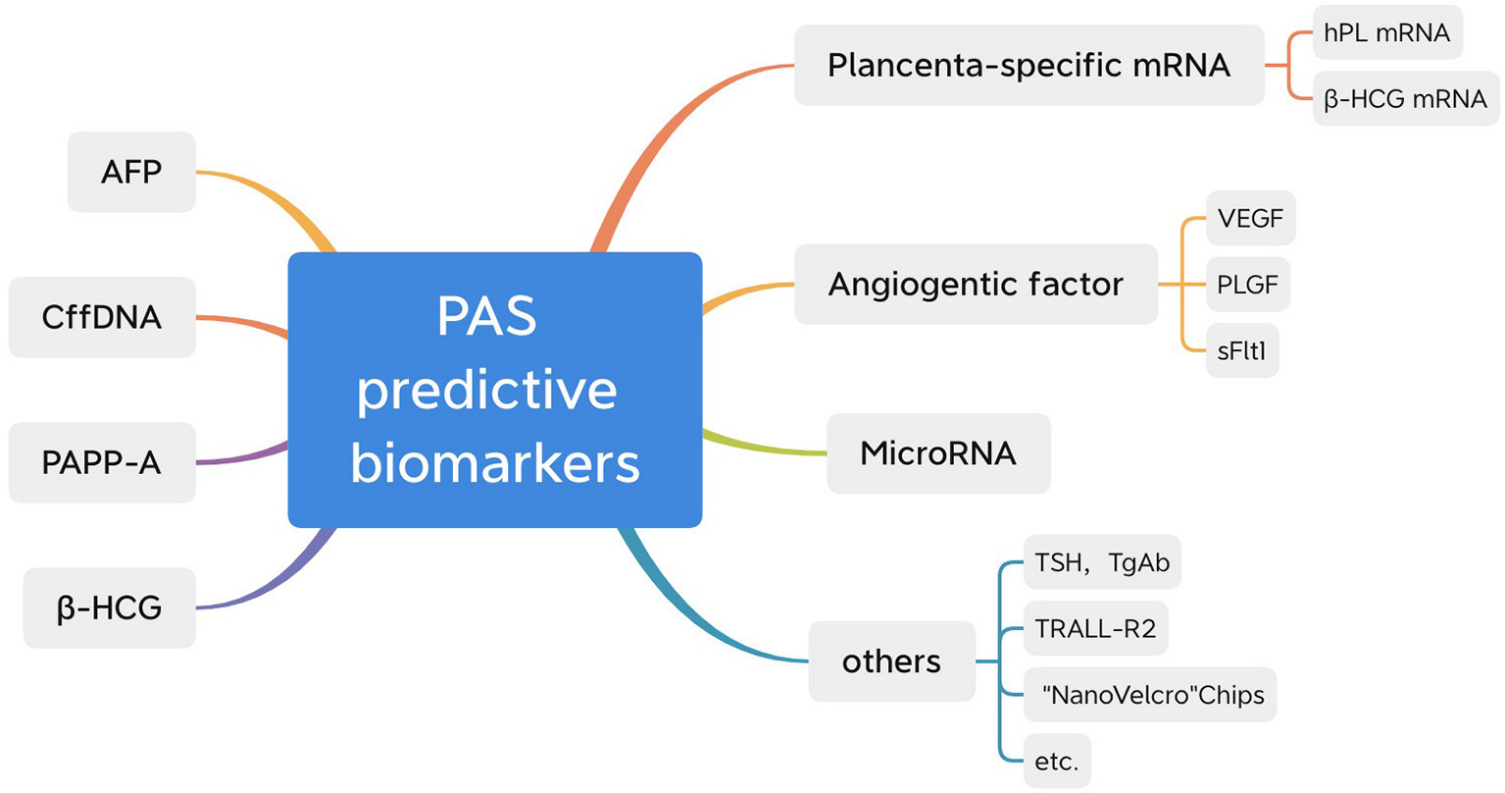
Biomarkers related to PAS.

This study reviewed the existing literature and summarized around 10 potential biomarkers to assist in the prenatal diagnosis of PAS (Figure 1). Some of them, such as β-hCG, AFP, cffDNA, and PAPP-A, have already been widely applied in clinical practice for other purposes. As PAS is a new domain and its morbidity is still low, scientists have yet to produce comprehensive research. Though we have already known that they are associated with PAS, more convincing published results will accelerate the application of such biomarkers. Little research exists on other newly reported biomarkers, such as microRNAs, placenta-specific mRNA and NanoVelcro chips; however, they are more specific and less influenced by other complications. The most significant hurdle is the cost of testing technology. With more thorough research, these biomarkers may have a bright future. According to previous studies, combining certain biomarkers with ultrasounds may improve the accuracy of a PAS diagnosis. In a study of cell-free placental mRNA, Behery et al. found that all six of the false-positive cases diagnosed by ultrasound and color Doppler in their study were from the group confirmed without placenta accreta, with an insignificant rise in cell-free placental mRNA levels (27). Furthermore, NanoVelcro chips can improve ultrasound accuracy. Thus, based on the current research, these two biomarkers can be further explored in the future. In addition, the current study about PAS complications is mostly related to hemorrhage and hysterectomy. Some other complications, including newborns' health conditions, premature labor, bladder invasion, and influence on the next pregnancy, were rarely counted and discussed. More focus should be placed on these areas in the future.

Currently, prenatal Down syndrome screening is widely performed on the basis of second-trimester or first-trimester maternal serum markers in clinical studies. By testing a combination of maternal serum levels of AFP, uE3, HCG, and PAPP-A, doctors can screen for antenatal aneuploidy diseases, including Down syndrome, trisomy 18 syndrome, and neural tube defects (NTD). The sensitivity of the test is 60–70%. Except for uE3, other biomarkers have been associated with PAS. In this way, co-analyzed serum markers may be beneficial for the antenatal screening of PAS and could assess the risk of PAS disorders. A retrospective case-control study in Israel co-analyzed maternal serum AFP and HCG in the second trimesters. The AUC was 0.668 (95% CI 0.611–0.721, *p* < 0.0001), and the sensitivity and specificity were 63 and 64%, respectively. Thus, they distinguished a percentile MoM cut-off approach between two groups: a high-risk group [patients with AFP, HCG, or both above the 75th percentile, with an odds ratio (OR) for pathological placentation of 2.27, 95% CI 1.42–3.63] and a low-risk group (patients with AFP, HCG, or both below the 25th percentile, with an OR for pathological placentation of 0.38, 95% CI 0.24–0.60) (9). Therefore, according to AFP and HCG values, researchers can focus on patients at higher risk of PAS, which is convenient for doctors making prenatal diagnoses of PAS. Biomarkers of aneuploidy in PAS are re-listed in Table 2 in detail. Different studies have provided significantly different cut-off values. Only a few studies have explored the relationship between biomarkers and the prognosis of PAS. Thus, the best appropriate cut-off value in Chinese should be found, and extensive research must be conducted that includes APOs to find a relationship between biomarkers and the prognosis of PAS. Few studies have researched co-analyzing biomarkers, and most of them only researched two biomarkers. Similar to an aneuploid prenatal diagnosis, researchers must explore whether a better combination of biomarkers exists for predicting the type of PAS and the prognosis of PAS. Once again, a more appropriate cut-off value must be found for the Chinese. Once we finish these series of research, they are easy to be wildly used in clinical. Their mature detection methods and affordable prices greatly reduce our obstruction and save our research time.

**Table 2:** Biomarkers of aneuploidy in PAS.

**Marker**	**Study**	**Trimester**	**Gestational weeks**	**Total case**	**Cut-off value**	**Sensitivity**	**Specificity**	***P***	**Predict the prognosis**
AFP	Oztas et al.	second	16–20w	316	1.25MoM	0.8594	0.7143	=0.036	Y
	Berezowsky et al.	second	16–19+6w	301	0.99MoM	0.71	0.46	<0.027	Y
β-hcg	Thompson et al.	first	11–13w	560	0.81MoM	–	–	=0.061	N
	Buke et al.	first	–	88	1.42MoM	–	–	=0.042	N
	Berezowsky et al.	second	16–19+6w	301	1.25MoM	0.53	0.68	<0.0001	Y
PAPP-A	Thompson et al.	first	11–13w	560 (33AIP)	1.40MoM	–	–	=0.002	N
	Lyell et al.	first	10w 0d−13w 6d	736 (37AIP)	2.63MoM	–	–	–	N
	Penzhoyan et al.	first	11w 0d−13w 6d	87 (25AIP)	1.30MoM	–	–	=0.640	Y

*N, No; Y, Yes*.

Today, many pregnant women have prenatal examinations in basic medical institutions without the necessary clinical requirements or experienced doctors. Therefore, some pregnant women with a potentially high risk of PAS may be misdiagnosed. This study suggests a new method for predicting PAS. First, all pregnant women should be divided into different risk groups for PAS. Second, follow-up ultrasounds and specific biomarker tests should be performed for not-low-risk pregnant women to guard against typical warning signs. Finally, a PAS scoring system should be created to predict PAS prognosis, which would provide exceptional convenience for clinical work.

## Conclusion

Although this study was devoted to the antenatal diagnosis of PAS, up to 50% of pregnancies with PAS go undiagnosed before delivery in clinical practice, which results in increased morbidity. Today, ultrasound is still the first-line detection method for PAS. Summarizing the published literature revealed eight biomarkers in maternal serum that could be used to predict PAS in the future. However, how these biomarkers predict the severity of PAS and their relationships with PAS complications are still not fully understood. Some suggestions for increasing the accuracy of diagnosing PAS have been proposed and must be further explored. With the third-child policy carried out in China, the number of women becoming pregnant again after a cesarean section will increase significantly, which will subsequently increase the incidence of PAS. Therefore, it is important to identify PAS early and make accurate risk stratification. Applying this process in clinical settings will benefit countless families.

## Author Contributions

SW generated the idea. TZ wrote the article. Both authors contributed to the article, revised the manuscript, and approved the submitted version.

## Conflict of Interest

The authors declare that the research was conducted in the absence of any commercial or financial relationships that could be construed as a potential conflict of interest.

## Publisher's Note

All claims expressed in this article are solely those of the authors and do not necessarily represent those of their affiliated organizations, or those of the publisher, the editors and the reviewers. Any product that may be evaluated in this article, or claim that may be made by its manufacturer, is not guaranteed or endorsed by the publisher.
